# Long-Term Care Residents’ Experiences of Telemedicine – Video Consultations With Their Regular Physician in Rural Northern Sweden

**DOI:** 10.1177/21501319261477002

**Published:** 2026-08-18

**Authors:** Lina Ärlebrant, Anna-Karin Hurtig, Anette Edin-Liljegren

**Affiliations:** 1 Department of Epidemiology and Global Health, 8075 Umeå University, Umeå, Sweden; 2 Swedish Centre for Rural Health, 8076 Region Västerbotten, Storuman, Sweden

**Keywords:** eHealth, long-term care, telehealth, qualitative, rural, video consultations, remote consultations

## Abstract

**Introduction:**

Demand for long-term care is increasing globally due to ageing populations and workforce shortages, particularly in rural areas. Video consultations (VC) may support interactions between residents and healthcare professionals and gained prominence during the Covid-19 pandemic. However, research on long-term care residents’ experiences of VC remains limited, and ethical challenges have been highlighted. This study aims to explore residents’ experiences of VC.

**Methods:**

A qualitative study with an inductive approach was conducted. Interviews (n=10) were carried out with long-term care residents aged 59–96 who had used VC with their designated physician, across three rural municipalities in northern Sweden. Data were analysed using thematic analysis.

**Results:**

Two themes and six subthemes were identified: (1) Emotional Experience of being Connected yet Apart, and (2) Conditions that Enable, Shape, and Separate.

**Conclusion:**

Experiences were shaped by individual preferences and contextual factors rather than age, with physician behaviour playing a central role. Although attitudes towards VC are becoming more positive, it should remain a complement to face-to-face consultations. Risks such as reduced autonomy, privacy concerns and technical limitations must be addressed. Familiar providers and tailored solutions may enhance autonomy, while VC may also foster courage and empowerment.

## Introduction

A longer life is an exceptionally valuable resource and the outcome of a collective achievement in improving health worldwide.^1^ The pace of population ageing is accelerating and by 2050 the number of people >65 years worldwide is projected to more than double, with a percentage change of 120% between 2019 and 2050.^2^ With increasing age, individuals are more likely to develop multiple chronic conditions, which are also associated with higher levels of health care utilisation.^3^ Information and communication technologies (ICT) are believed to be an essential factor in ensuring universal health coverage for individuals of all ages.^4^ Telehealth uses ICT to deliver health care services where distance separates patients and providers, improving access to quality health services. This is seen as particularly valuable for ageing populations living in rural areas.^5^ In the second action plan of the *WHO Global strategy on ageing and health*, national and international partners are promoted to act and develop digital technologies that contribute to good-quality long-term care services.^1^

Synchronous telemedicine, such as video consultations (VC) is a promising solution particularly for managing chronic conditions, as it provides access and continuity of care, and ability to assess patients visually.^6^ When investigating VC interventions for older adults, quantitatively, VC was shown as equivalent or superior to face-to-face (FTF) meetings in the management of chronic conditions, and reported high patient satisfaction and no significant problems adapting to VC. Moreover, VC was preferred compared to FTF meetings due to its conveniences.^7^ However, technological literacy and absence of smart devices for older adults could hinder the effectiveness,^6,8-10^ as well as difficulties with hearing problems and the inability of physical assessments.^7-11^

Globally, there is a growing need for long-time care services due to ageing populations, presenting challenges in workforce shortages and reduced supply of informal caregivers while maintaining affordability and quality of care.^12^ Nursing homes, or residential aged care facilities, play a vital role in the continuum of healthcare services across the globe to provide long-term care for individuals, primarily older adults, who require assistance with daily activities and medical supervision that cannot be adequately provided at home.^13^ Older adults in rural areas are particularly vulnerable to adverse outcomes due to complex medical needs, including multimorbidity and involvement with multiple providers. Thus, combined with limited access to specialist care, at a time when digital technologies are increasingly implemented in healthcare despite the absence of comprehensive guidance.^14^ The nature of encounters between patients and healthcare professionals have changed with increasing use of technology such as VC.^15^ Covid-19 highlighted vulnerabilities in long-term care settings,^16^ re-emphasising the importance of VC in supporting interactions between long-term care residents and providers.^17^ A post-Pandemic analysis showed that between 2019 and 2023 there was a remarkable global effort to utilise VC and European countries are at the forefront of this.^18^ Despite the extraordinary increase in use, many patients still prefer FTF consultations,^9,19-21^ and research found two key barriers to use digital technologies in healthcare, such as VC, perceived by the older adults; 1) negative attitudes and technology anxiety, and; 2) lack of trust − affecting both adoption and usage.^22^ Most of those barriers is believed to occur at the pre-adoption stage and reasons why older adults choose, or are unable to adopt goes beyond the specific technology, addressing complexities in inequal social and economic conditions.^22,23^ Various forms of trust are influencing older adults’ use of technology in healthcare, such as trust in the provider, and trust in patient safety.^24^

Research exploring long-term care residents’ experiences of VC is lacking.^17,25^ However, the perspective of healthcare staff has been explored, indicating that VC adds a small value compared to e.g. sending photos, but is somewhat less time consuming than other communication channels, and most useful for clarifying urgent problems.^26^ There are mixed views on the effectiveness of VC in nursing homes among physicians suggesting that VC may not be a suitable substitute for most FTF services.^27^ Moreover, healthcare providers experience telemedicine to enable care quality improvement with remote specialists but technical problems reduce the ease of use.^17^ A literature review on healthcare providers' experiences of telemedicine in long-term care concludes that residents' experiences must be included in future studies.^17^ The effect of telehealth versus traditional care for home care patients with long term conditions has been investigated. A systematic review studying a population with the mean age of 71 years old found no difference between telehealth and traditional care regarding outcomes as quality of life, psychological wellbeing, and physical function but some participants fear isolation and reduced FTF contact.^28^ When focusing on nursing home residents' perspectives on VC, investigating the ethical acceptability, one study found various ethical challenges related to autonomy, participation, privacy, self-conception, beneficence, security, and justice, suggesting further studies on residents' perspective of VC.^25^

Conducting research in rural areas today provides an opportunity to study the demographic structures that many densely populated cities will face in the future. In this study’s setting, some of Sweden’s most sparsely populated municipalities is found, and the proportion of people over 65 years today is 29%, compared to 20.1% at the national average.^29^ Rural northern Sweden is in the forefront, introducing telemedicine solutions in the early 1990’s, and having a head start due to the demographic shift. Research-based evidence and experiences from these settings are therefore incredibly valuable in addressing the gap in long-term care residents’ experiences with VC in healthcare and in advancing sustainable healthcare solutions globally.^30^

The aim of this study is to explore long-term care residents’ experiences of video consultations with their regular physician in rural northern Sweden.

## Methods

A qualitative study design with an inductive approach was utilised to explore the experiences of patients in meetings with their physician via VC. The Consolidated Criteria for Reporting Qualitative Studies (COREQ) were followed to report this study,^31^ and the ISSM COREQ Checklist is attached as Appendix 1.

The study was conducted in three municipalities in Västerbotten County, Northern Sweden. The municipalities are responsible for the healthcare services to individuals residing in nursing homes, and homecare in ordinary housing – in this study referred to as long-term care residents. The healthcare is delivered by registered nurses (RNs), physiotherapists, and occupational therapists. The municipalities in Sweden (n=290) are responsible for nursing and rehabilitation but not medical interventions that require a physician, since this is the responsibility of the region (n=21).^32^ However, the Patient Act in Sweden state that the patient should have access to a designated physician within the primary care system where they are listed.^33^ In the studied setting, routine medical care for individuals entitled to municipal healthcare according to a formal needs assessment decision, is delivered exclusively via scheduled VCs, whereby their designated physicians conduct their rounds remotely in collaboration with on-site RNs. The VC is held in the resident’s home in a nursing home or ordinary housing, together with the RN on site and their designated physician connecting and visible on the RNs laptop. These physicians are employed by the region and connecting from sites in Sweden and Spain. In case of emergency, patients are transferred to the primary care unit in the area. The three municipalities in this study have a population density of 0.3-0.8 inh/km^2^,^34^ and are classified as sparsely populated areas,^35^ with long distances to hospitals.^32^ Participant characteristics are shown in Table 1.

**Table 1.** Participant Characteristics (N = 10, Coded as P1-P10)

Characteristic	Value
**Age**	59-96 years (Median=83)
**Female sex**	4 (40 %)
**Born and raised in Sweden**	10 (100 %)
**Residing in nursing home**	6 (60%)
**Self-reported digital competence**	
High	1 (10 %)
Intermediate	3 (30 %)
Low	6 (60 %)

Participants were recruited by the RNs working at the nursing home and in home healthcare using purposive sampling. Inclusion criteria were: adults (>18 years) receiving municipal healthcare in nursing homes, or in ordinary housing; Swedish speaking; participated in one or more VC with their regular physician as part of planned (non-acute) care; and able to communicate the experience. The patients were informed about the study and its voluntary participation by the RN, verbally and in written information from the researchers. The information letter also contained a short presentation of the first author (Appendix 2). The RNs contacted the first author with contact details from patients willing to participate and the participants gave written consent to participate in the study. One patient declined participation in the study and expressed a wish to meet the physician FTF. The sample size reflects the sparsely populated area since all patients having a VC during this study were asked to participate, resulting in 10 interviews. Moreover, the sample size is motivated using the concept “information power”, since this is a qualitative interview study, the study aim was narrow, and the participants had characteristics specific for the aim.^36^

The 10 semi-structured individual interviews were held in the participants home by the first author from June 2024 to March 2025 and audio recorded. There was no other person present during the interview and in case of interruption, the recording was paused. The interview guide included background questions about country of birth and digital competence, but focused on the experience of involvement, continuity, security, and good quality healthcare – in relation to the VC. It had previously been used in another study setting (Appendix 3).^37^ The length of each interview varied from 17−54 min (Mean=38min) and was transcribed using noScribe (v0.6), and the Swedish national research infrastructure Språkbanken and SweClarin, followed by a manual review by the first author to ensure verbatim transcripts. The sample size was discussed by the authors using the concept of “information power” rather than “data saturation”, as recommended for studies employing qualitative thematic analysis.^36^ Since the study aim was narrow and the participants had characteristics highly specific to that aim, the information power was considered sufficient despite the relatively small sample size.

The data analysis was carried out by the first author in consultation with the co-authors, using thematic analysis as described by Braun & Clarke,^38^ and MAXQDA software (Version: MAXQDA 24 (Release 24.10.0)). The authors read the transcripts repeatedly to familiarise with the data and inductively coded one transcript independently to look for common patterns. The coded transcript was discussed between the authors who reached a joint idea of collating codes into themes. The first author continued the process of analysis with moving back and forth between the entire transcripts, inductive coding, and identifying themes. Themes and codes were evaluated several times with the co-authors and derived by combining and comparing codes related to one another, covering all of the coded data, while closely linked to the original data. The review of codes finally identified two common perspectives separated by different levels of experience – such as emotional and technical – which represented the themes. The study was approved by the Swedish Ethical Review Authority (Dno.2024-00948-02).

## Results

Data analysis resulted in two themes offering a multi-dimensional perspective through which patients’ experiences of VC can be understood: Theme 1. Emotional Experience of being Connected yet Apart; Theme 2. Conditions that Enable, Shape, and Separate. The themes and associated 6 subthemes are presented in Table 2. In the body text, subthemes are indicated in bold under each theme presented below. The supporting quotations from residents have been translated from Swedish into English using DeepL (Free version 26.21).

**Table 2.** Themes and Subthemes

**Emotional Experience of being Connected yet Apart**		
Remarkably similar	Emotion was shaped by behaviour and familiarity	Missing physical presence with detaching screens

**Conditions that Enable, Shape, and Separate**		
The home and nurse on site facilitate assertiveness	Video image increase access	Limitations without physical presence

### Theme 1. Emotional Experience of being Connected yet Apart

The emotional experience is described in feelings aroused during VC and in how the physician’s behaviour influences the emotions. In many cases this is reflected upon in comparison to traditional care FTF recognising both opinions of VC as being just the same, and of what patient’s feel are missing.

When VC is experienced as being **remarkably similar** compared to FTF consultations patients express that VC, quite simply, went well and are not always able to elaborate more in-depth descriptions on why. It is referred to as feeling no difference and being able to contribute and participate as usual. The well-functioning VC was, however, somewhat surprising and unreal for some. Others had more of a matter-of-fact attitude, perceiving it as nothing particularly strange.

*“There’s really no difference. Because you see people talking. And we’re used to watching TV too. And it makes a big difference that we’re used to seeing people talk on TV. We learn certain things through that. There’s a lot we’ve learned from television.”* (P8)

Overall, the **emotion was shaped by behaviour and familiarity**, which is stated with examples of how some physicians make patients feel relaxed and able to talk about anything. Whereas others make patients feel ignored and that they don’t get along. When the interaction during VC was positive the patient’s own behaviour changed in terms of listening carefully, asking questions again to understand, and willingly share their feelings. Contributing to a good atmosphere was physicians smiling and joking around but also showing interest by asking questions. It was important that the physician was keen to talk things through, focusing, and acting to help the patient. That behaviour made patients feel encouraged, satisfied and taken seriously. To be listened to increase the sense of trust and safety, and was most important for some.

*“The positive thing is, I’m not against this thing of talking to a physician on video. ‘Cause I’ve kind of experienced how good it can be. But also how it’s not good. So I’ve got that to compare with. But it’s about personalities, of course.”* (P1)

A familiar physician is often preferable during VC and seen as important to make the experience as good as FTF, at least if the physician speaks an understandable language without a strong accent. The physician is as important as family for some, making them feel safe, and they would rather travel than meet an unknown physician on VC. A known physician also makes patients feel ok about showing their home over video. There is, however, also opinions showing that familiar physicians do not matter since some felt contact and trust via VC even if they did not know each other and had never meet before. Some state that they are seeing a lot of different physicians now, compared to when they met on site, which is experienced as bad and as VC is not, by itself, contributing to continuity.

*“It depends really. If it’s a doctor I know, someone I’ve seen before. Otherwise, I’d rather go through the door at the health centre than talk to someone whose name I barely know…Yes, but I mean, if it’s like… I don’t mind going to the health centre, but if it’s someone I know – a doctor who knows me – then talking on the screen works just as well.”* (P6)

Preparation and information matter for the VC because it is experienced as difficult for some to express feelings over video but believed to be easier when you are prepared. Some knew about the VC ahead, and for some there was no time to think because the RN just turned up with the computer. Telling how they feel contributes to the planning of their healthcare, patients explain. It is preferable to know and be promised the possibility to meet a physician FTF if needed– something that should be offered occasionally no matter what. There is some uncertainty expressed in connection to if, and when, they can meet their physician FTF. But also an understanding that VC saves time and money.

*“But I find it hard to answer. It’s difficult to answer. When I’m not really in my own body, so to speak, then it’s hard to say. “How are you?” Well, what do you mean, how am I? What?! (I: It’s not easy to put your finger on it.) But it’s really hard. Really hard. So I kind of think through what I’m going to say to him. To sort of know in advance…I plan ahead, I do tell the doctor how I’m feeling.”* (P1)

Some felt they were **missing physical presence with detaching screens** and expressed it as losing a dimension and the connection with the physician. This is described as feeling different, as something else, and not quite the same. There is also a sense of resignation and incomprehension over the screens as the only thing offered. Some felt that it was impossible to create a bond with the physician over VC and were missing the physical contact and the ability to be examined properly. A wish to meet FTF instead was reported, and that VC should only be seen as a complement, not a replacement to FTF consultations. However, many reflected upon this as a habit that could possibly change over time, meaning that VC could be a more positive experience in the future. Others could not see any advantages with VC.

*“Well, it’s precisely that − something feels missing. But I’m not sure if it’s just a matter of habit, I really can’t say. I mean, we’ve always been used to seeing the physician in person, and maybe that’s made it a kind of routine. So, you end up feeling the same way, I don’t know.”* (P9)

Some experienced that the screen enables detachment compared to meetings FTF. The screen between them was seen as the reason why some experienced the physicians could more easily distance themselves, becoming ignorant and not taking responsibility. This made patients feel not listened to, resulting in physicians pushing through decisions without agreement − “*which is not ok*” − and made VC a bad experience overall.

*“But it seems that (the physicians) I meet through the screen want to keep their distance, so they don't have to take any responsibility or anything like that. And then you feel a bit… They end up making decisions that aren’t very wise.”* (P6)

### Theme 2. Conditions that Enable, Shape, and Separate

The experience of VC is influenced by contextual factors and technical functionality, such as being at home, the nurse on site, physical presence, sound and image – all contributing or hindering the interaction with the physician. Depending on these conditions’ patients experience VC suitable depending on the nature of issues.

**The home and nurse on site facilitate assertiveness** by making some feel more confident and empowered in their home, also described like having a closer contact with the physicians via VC. It was a more focused conversation between the patient and the physician, directly, and with eye contact – like if the physician was sitting by their kitchen table, close. This resulted in patients saying things they wouldn’t do FTF and feeling included rather than left out even when the RN sometimes spoke to the physician.

*“I would say it works better. Being in your home environment makes you feel more confident and bolder. (I: Why do you think that is?) Well, that’s just how it is. I think video consultations are a good solution. It’s like you act in a different way compared to being physically present at the health clinic. Yes, I really think so. For example, I said, “You really need to leave that medication alone,” when someone wanted to discontinue it. That’s not something I would say in a traditional healthcare setting. But here, I can say it.”* (P7)

The nurse on site is crucial for the VC to work, because of unfamiliarity of the technic and the need for more explanations on occasions. Most often mentioned is the need of help from the RN with the audio regulation because of physicians talking rapidly or an unsatisfactory sound. But also, for preparation and introducing each other, sometimes joking around, and making the atmosphere relaxed. RNs also assist with writing down information given during VC for patients preferring that to remember. This kind of help from RN is described as great and contribute to safety.

*“Yes, no, there really must be a nurse there… someone who can make sure you hear things properly. Yes, if you’re young and healthy then it’s no problem. But it was a bit hard to hear sometimes. Yes, sometimes we can be a bit…And then the nurse said, she said what was being said. Oh yes, it worked perfectly. So, there must be a nurse sitting next to you. (I: Yes, then it works?) Yes. Then it works. Absolutely. I think it works well that way.”* (P10)

Some mentioned that **video image increase access** and that it is positive not to travel. Talking with the physician from the bed in the future would be practical according to some. One patient had experienced VC during an acute event and got the help needed even then.

*“Not having to travel 150–200 kilometres to see a doctor and sit down and talk—that’s a relief. First of all, you’re so tired after that trip. And… you get him right there in your living room. It’s just perfect. I think that’s how it will be in the future.”* (P1)

Additionally, patients experience advantages of video image, since they felt it was possible to express themselves in a good way and ask the questions they wanted during VC thanks to the sufficient video image. Facial expressions were mentioned as important to perceive, which was possible, as it was also noted that physicians could recognise patients’ needs from their point of view. In comparison with telephone VC was advantageous because of visibility making for example, an accent easier to understand. Overall, the quality of the video image was satisfactory.

*“(I: It hasn’t felt difficult to talk?) No, no. On the contrary, I found it easy to talk.”* (P7)

*“(I: Was it harder to see on the screen than when we sit like this?) No, no. The image itself worked well.”* (P3)

There are times when patients experience **limitations without physical presence**, as when physical assessments or laboratory test are required, and in cases involving for example cut wounds. Other situations mentioned are when in pain, because it is seen as best assessed in the same room. VC is working well for simple issues, like to check how you are doing, or for a medication review.

*“No, but it really depends on what it is. You can’t really treat things with a computer. No. Unless it’s something about medication or something like that. Something simpler. (I: So it depends on what it’s about?) Yes, it depends on what it’s about. That’s how it is. I think this works well for these symptoms, yes.”* (P10)

Patients describe that the sound quality is crucial and can be insufficient because of hearing loss or technical limitations resulting in a negative experience of VC. It was described as if the sound was to high pitched, too loud, or too quiet. On one occasion, the physician was hoarse, which led to difficulty hearing what was being said. The sound quality is seen as crucial to avoid misunderstandings during VC.

## Discussion

This qualitative study highlights long-term care residents’ experiences of VC with their physician in rural northern Sweden and are presented in two themes describing different perspectives. First, the emotional experience of VC; second, the conditions affecting the experience. Our findings contribute to closing the knowledge gap regarding residents’ experiences of VC in their own words, which is valuable for understanding the development and implementation of VC that could help expand universal healthcare access, especially in rural areas.

Some residents perceived VC as nothing particularly strange and considering it as familiar as FTF consultations or watching television. This may be surprising given the commonly assumed negative attitude toward technology among older people,^39,40^ and previous research have stated preference of FTF consultations.^9,19-21,25^ Our result is, however, in line with recent research showing that attitudes on technologies differs within generations and are not necessarily depending on age, but might be more related to individual experieces.^41,42^ Moreover, previous experience or interest is – rather than old age, described as a reason not to use telehealth.^43^ Findings from a national survey conducted in Sweden published 2023 state that attitudes towards technologies for active and healthy ageing are shared across generations in many ways, but notable differences were e.g. that the oldest generation (70-79 years) perceived ICT products, such as VC, as significantly less useful and time-saving compared to the youngest generation (30-39 years).^44^ Consistent to our findings indicating a change of habits towards a more positive experience in the future, most of the barriers against digital platforms for older persons is indicated to occur at the pre-adoption stage, and only a few exists post-adoption.^22^

Importantly, some residents perceived VC as limiting, as the absence of physical physician presence hindered the development of a meaningful relationship. These perceptions were influenced by physicians’ behaviour during VC, while the screen itself was seen as creating distance. A systematic review studying electronic mental health interventions, including VC, among older adults found building trusting relationships and providing emotional support as needed from the healthcare providers.^40^ Since “trust is a patient’s readiness to put their health and privacy in the hands of a telemedicine service provider”, it plays a crucial role in ensuring the elderly a satisfactory outcome.^24^ The challenges with provider-patient relationships during VC is well known from previous research.^45-47^ One way to strengthen the positive experience of VC, found in our study, is to cherish the relationship by showing interest, be focused, and keen to talk things through, but also taking time to joke around.

Deployment of homecare technology has introduced serious concerns regarding autonomy of the individual – which is recognised and valued as an ethical approach of caring.^48^ Systems that honour patients autonomy in healthcare decisions and are adapted to home settings is needed.^48^ Our finding highlights neglected autonomy when residents experienced the screen as the only option offered, sensing resignation, uncertainty, and incomprehension. VC’s negative impact on patients’ perceived autonomy has been noted in a systematic review evaluating VC in Primary Care.^49^ Personal autonomy is believed to influence the acceptance of technology.^42^ However, technology is sometimes forced on older adults without considering their willingness to use such devices, which may lead to a less optimal long-term adoption.^20,41^ Patients feeling forced to use VC, or feeling like their only option is VC, may negatively impact opinions of VCs and their feelings of voluntariness.^20^ In consistence with other research, residents state VC as only a complement, not a replacement to FTF consultations,^10,11^ and prefer certainty for FTF consultations with the physician if needed. This is crucial to consider regarding residents’ experiences of VC with healthcare. However, due to limited resources in the healthcare systems globally, it is necessary to set priorities. Our results highlight that VC provides satisfactory experiences in many cases, allowing FTF consultations when necessary to maintain security and high-quality healthcare. One way of increasing patients’ autonomy despite limited resources, is to place greater value on preparation and information, as needed from the resident’s perspective. Knowing that FTF consultations are available appears to be highly important. Preparation guidance for VC from the healthcare professionals perspective is found in previous research but should be further investigated and formulated for patients.^50-53^

When integrating VC in long-term care, residents’ home is the space for the consultation. This has been shown in research to bring both possibilities – such as a strengthened patient empowerment, and hinders – such as increased vulnerability.^11^ Our findings state home as an enabling condition because residents felt more confident and could say things they would not do FTF during VC. Similarly, when describing patients’ experiences of receiving care in their private homes, self-esteem was empowering the patient’s, maintaining self-determination in their own homes.^54^ Notably, nursing home residents perceptions of VC also indicate a reduction in privacy when the care facility is the resident’s home and VC is held together with a nurse on site.^25^ Consistent with our findings, residents state an absolute need of having a provider on site to manage the VC, at the same time describe providers as almost family members.^25^ These findings could indicate VC at home to strengthen residents’ confidence and empowerment, facilitated not only by their home environment, but also due to familiar providers. Research suggests continuity as not a universal need, but a conditional one, especially relevant for patients with chronic and long-term conditions.^55^ Problems with hearing has been highlighted as hindering older persons during VC in several studies,^7-11,43^ in consistence to our findings. The nurse on site was a critical enabler, increasing the quality of VC, helping with audio regulation and more explanations when needed. Thus, while residents are depending on assistance during VC, consultations with the physician in private should also be offered, as noted in previous research.^25^

The experiences of VC have seldom before been explored through interviews with long-term care residents themselves. This study has the strength of performing interviews with a wide age-group of participants from different areas conducted by a nurse specialized in elderly care with long experience of long-term care in rural areas, interviewing, and in qualitative research methods (first author). This contributes to the credibility adding possibilities to capture a broader spectrum of experiences and is further strengthen with the analysis process of repeated discussions among the authors, as well as reporting by using quotes, and according to QOREQ.^31^ There is a risk of bias using RNs caring for the residents in recruitment which could lead to a more positive result. However, our findings state negative experiences of VC which argues against a biased recruitment. The sample comprised 10 patients, which may be considered small and a limitation of the study. However, this should be understood in relation to the context characterised by a very low population density, a generally low frequency of home visits – in this case VC – in long-term care, as well as the need for participating residents able to recount one’s experiences in an interview. The authors consider the sample to provide sufficient information power for this study. However, the purposive sampling does not show representation of the residents in long-term care in general, since the inclusion criteria excluded persons unable to remember the VC or participate in an interview. This could have been more thoroughly investigated with an observational study that could include persons with cognitive impairment, not able to participate in an interview. Future research should continue exploring long-term care residents’ perspectives of VC to fil the knowledge gap, especially investigating the impact on experience and outcomes regarding ways to strengthen residents’ autonomy, individualise VC in healthcare, and limit technical problems with e.g. sound quality.

## Conclusions

The experience of VC is not a matter of age but relates to individual preferences and complex conditions, not least the physician’s behaviour behind the screen. It is important for the relationship and atmosphere during VC to take proper time and to show interest. The attitudes towards VC among residents could be shifting towards more acceptance. For long-term adoption and a satisfactory VC experience, FTF consultations must remain available to ensure VC complements rather than replaces FTF care. Further, the risk of decreased autonomy and privacy must be taken seriously. As should the often unsatisfactory sound quality in VC. Familiar providers are preferable and individualized solutions, preparation, and information could increase autonomy. Findings suggest a positive impact on residents’ courage and empowerment, which could be important advantages to protect in future implementations of VC in healthcare. Key clinical implications are outlined in Box 1.

**Box 1.**
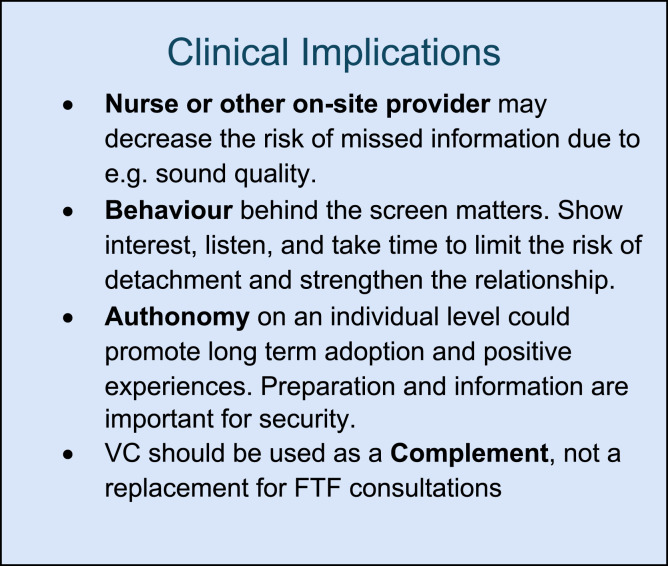
Clinical Implications

## Supplemental Material


Supplemental Material - Long-Term Care Residents’ Experiences of Telemedicine – Video Consultations With Their Regular Physician in Rural Northern Sweden
Supplemental Material for Long-Term Care Residents’ Experiences of Telemedicine – Video Consultations With Their Regular Physician in Rural Northern Sweden by Lina Ärlebrant, Anna-Karin Hurtig and Anette Edin-Liljegren in Journal of Primary Care & Community Health.

Supplemental Material - Long-Term Care Residents’ Experiences of Telemedicine – Video Consultations With Their Regular Physician in Rural Northern Sweden
Supplemental Material for Long-Term Care Residents’ Experiences of Telemedicine – Video Consultations With Their Regular Physician in Rural Northern Sweden by Lina Ärlebrant, Anna-Karin Hurtig and Anette Edin-Liljegren in Journal of Primary Care & Community Health.

Supplemental Material - Long-Term Care Residents’ Experiences of Telemedicine – Video Consultations With Their Regular Physician in Rural Northern Sweden
Supplemental Material for Long-Term Care Residents’ Experiences of Telemedicine – Video Consultations With Their Regular Physician in Rural Northern Sweden by Lina Ärlebrant, Anna-Karin Hurtig and Anette Edin-Liljegren in Journal of Primary Care & Community Health.

## Data Availability

The interviews are related to person’s health and therefore personal data. The setting of data collection is sparsely populated areas which makes data particularly sensitive with a risk of identifiable information. Therefore, data from this study is not publicly available.*
