# Supplementary material for: Long-Term Care Residents’ Experiences of Telemedicine – Video Consultations With Their Regular Physician in Rural Northern Sweden
Source: J Prim Care Community Health. 2026 Aug 18;17:21501319261477002. doi: 10.1177/21501319261477002 (PMC13487111; doi:10.1177/21501319261477002)
Supplement: Supplementary file 1 [file sj-pdf-1-jpc-10.1177_21501319261477002.pdf]

Appendix 1. Reporting of method following COREQ (COnsolidated criteria for REporting Qualitative research) Checklist

Topic		Item No.	Guide Questions/Description	Report and Page No.
	**Domain 1: Research team and reflexivity**			
	Personal characteristics			
Interviewer/facilitator		1	Which author/s conducted the interview or focus group?	Page 3. First author
Credentials		2	What were the researcher's credentials? E.g. PhD, MD	Title Page.<br>First author (1) PhD student, MSN<br>Co-authors: (2) PhD, MD (3) - PhD
Occupation		3	What was their occupation at the time of the study?	Title page.<br>(1)- PhD student and project coordinator<br>(2)- Professor<br>(3)- Adjunct associate professor and a research-strategist
Gender		4	Was the researcher male or female?	Female researchers
Experience and training		5	What experience or training did the researcher have?	Title page<br>(1)- Is a registered nurse specialized in elderly care experienced and educated in interview technic and qualitative methods. Living in a rural area in Västerbotten County.<br>(2)- Is trained as a medical doctor. She has experience in health systems research and a particular interest in community health systems across different contexts. Living in an urban area of Västerbotten County.<br>(3)- Has extensive knowledge and experience in qualitative research methods and the rural context. Living in a rural area in Västerbotten County.
	Relationship with participants			
Relationship established		6	Was a relationship established prior to study commencement?	Page 4 and Appendix 2.<br>The first author was introduced in the information letter before the interviews.
Participant knowledge of the interviewer		7	What did the participants know about the researcher? e.g. personal goals, reasons for doing the research	Page 4 and Appendix 2.<br>The information letter also contained a short presentation of the first author shown in Appendix 2. Information Letter
Interviewer characteristics		8	What characteristics were reported about the inter viewer/facilitator? e.g. Bias, assumptions, reasons and interests in the research topic	Appendix 2. Information letter.
	**Domain 2: Study design**			
	Theoretical framework			

Appendix 1. Reporting of method following COREQ (COnsolidated criteria for REporting Qualitative research) Checklist

Methodological orientation and Theory	9	What methodological orientation was stated to underpin the study? e.g. grounded theory, discourse analysis, ethnography, phenomenology, content analysis	Page 3. A qualitative study design with an inductive approach was utilised to explore the experiences of patients in meetings with their physician via VC. The data analysis was carried out by the first author in consultation with the co-authors, using thematic analysis as described by Braun & Clarke.
Participant selection			
Sampling	10	How were participants selected? e.g. purposive, convenience, consecutive, snowball	Page 4. Participants were recruited by the RNs working at the nursing home and in home healthcare using purposive sampling. Inclusion criteria were adults receiving municipal healthcare in nursing homes, or in ordinary housing, Swedish speaking, participated in one or more VC with a physician, and able to communicate the experience.
Method of approach	11	How were participants approached? e.g. face-to-face, telephone, mail, email	Page 4. The patients were informed about the study and its voluntary participation by the RN, verbally and in written information from the researchers. The RN contacted the first author with contact details from patients willing to participate. The participants gave written consent to participate in the study.
Sample size	12	How many participants were in the study?	Page 4. N= 10
Non-participation	13	How many people refused to participate or dropped out? Reasons?	Page 4. One patient declined participation in the study and expressed a wish to meet the physician FTF.
Setting			
Setting of data collection	14	Where was the data collected? e.g. home, clinic, workplace	Page 4. The 10 semi-structured individual interviews were held in the participants home by the first author from June 2024 to March 2025.
Presence of non-participants	15	Was anyone else present besides the participants and researchers?	Page 4. There was no other person present during the interview and in case of interruption, the recording was paused.
Description of sample	16	What are the important characteristics of the sample? e.g. demographic data, date	Page 4, Table 1.
Data collection			
Interview guide	17	Were questions, prompts, guides provided by the authors? Was it pilot tested?	Page 4. Appendix 3. The interview guide included background questions

Appendix 1. Reporting of method following COREQ (COnsolidated criteria for REporting Qualitative research) Checklist

			about country of birth and digital competence but focused on the experience of involvement, continuity, security, and good quality healthcare, in relation to the VC. It had previously been used in another study setting.
Repeat interviews	18	Were repeat inter views carried out? If yes, how many?	No
Audio/visual recording	19	Did the research use audio or visual recording to collect the data?	Page 4.<br>The interviews were audio recorded.
Field notes	20	Were field notes made during and/or after the interview or focus group?	No
Duration	21	What was the duration of the inter views or focus group?	Page 4.<br>The length of each interview varied from 17–54 min (Mean=38min).
Data saturation	22	Was data saturation discussed?	Page 4.<br>The sample size was discussed by the authors using the concept of "information power", since the method of analysis were qualitative thematic analysis, the study aim was narrow, and the participants had characteristics highly specified for the aim.
Transcripts returned	23	Were transcripts returned to participants for comment and/or correction?	No
**Domain 3: analysis and findings**			
Data analysis			
Number of data coders	24	How many data coders coded the data?	Page 5.<br>The three authors read the transcripts repeatedly to familiarise with the data and inductively coded one transcript independently to look for common patterns and a joint idea of collating codes into themes.
Description of the coding tree	25	Did authors provide a description of the coding tree?	Page 5, Table 2.
Derivation of themes	26	Were themes identified in advance or derived from the data?	Page 5.<br>The first author continued the process of analysis with moving back and forth between the entire transcripts, inductive coding, and identifying themes. Themes were evaluated several times with the co-authors.
Software	27	What software, if applicable, was used to manage the data?	Page 4,5.<br>Audio recordings were transcribed using noScribe (v0.6), and the Swedish national research infrastructure Språkbanken and SweClarin, followed by a manual review by the first author to ensure verbatim transcripts. MAXQDA software (Version: MAXQDA

Appendix 1. Reporting of method following COREQ (COnsolidated criteria for REporting Qualitative research) Checklist

			24 (Release 24.10.0)) was used for the data analysis.
Participant checking	28	Did participants provide feedback on the findings?	No
Reporting			
Quotations presented	29	Were participant quotations presented to illustrate the themes/findings? Was each quotation identified? e.g. participant number	Page 6-9
Data and findings consistent	30	Was there consistency between the data presented and the findings?	Page 5-9
Clarity of major themes	31	Were major themes clearly presented in the findings?	Page 5-9, Table 2.
Clarity of minor themes	32	Is there a description of diverse cases or discussion of minor themes?	Page 5-9
