# Supplementary material for: Long-Term Care Residents’ Experiences of Telemedicine – Video Consultations With Their Regular Physician in Rural Northern Sweden
Source: J Prim Care Community Health. 2026 Aug 18;17:21501319261477002. doi: 10.1177/21501319261477002 (PMC13487111; doi:10.1177/21501319261477002)
Supplement: Supplementary file 2 [file sj-pdf-2-jpc-10.1177_21501319261477002.pdf]

Appendix 2. Information letter

Would you like to participate in a study on digital healthcare appointments?

This document provides information about the project and what participation entails. Please read all the information before deciding whether to participate.

What is this project and why do you want me to participate?

In Sweden, regions and municipalities are working to improve healthcare, and the primary care reform ‘Good and Close Care’ advocates that healthcare be organized based on the needs and wishes of the population and patients. To make this possible, we need to find out what the wishes are and what conditions and resources healthcare has in different places. Rural areas and their inhabitants have different conditions than other parts of the country and therefore need to be studied specifically in order to provide all individuals in Sweden with equal and good healthcare.

In Västerbotten County, activities are underway to investigate whether it is possible to feel involved and secure with a physician who meets their patients remotely. The goal is to improve continuity in residents' healthcare contacts, strengthen trust in healthcare and increase access to physicians in rural areas.

You have been contacted because you have met your physician via video. We would like to hear about your experiences of this digital healthcare appointment and have obtained your name from the managers of the home care service, nursing home or the community hospital where you live.

The overall purpose of the project is to investigate the experiences of video consultations with your physician, with a focus on security, continuity and participation.

How will the study be conducted?

The study is designed as an interview study in which you will be asked to answer questions individually. If you choose to participate, you will be asked to sign a consent form to participate in the interview. Depending on what is possible for you, the interviews will be conducted in person or digitally via telephone or video. The time and place will be determined together with you.

The questions in the study concern the general need for care that you have, what is considered good care, and experiences of video consultations with your physician, with regard to factors such as participation, security and continuity. The interviews will be recorded digitally and the text will be transcribed verbatim and analysed systematically using scientific methods. The interviews are estimated to take 30–60 minutes. You can withdraw from the study at any time without giving a reason. To do so, please contact the researcher responsible for the study. If

you choose to withdraw after the interview has begun, only information that has already been analysed will be retained.

Picture of the researcher
Lina Ärlebrant

Hello!

My name is Lina Ärlebrant and I work at Umeå University. In this photo, you can see me in my "local village"-costume. I am a specialist nurse in elderly care but have been working in research for the past few years. I will be conducting the interviews for the study if you agree to participate.

Possible consequences and risks of participating in the study

Participating in the study does not entail any special advantages or disadvantages for you and does not affect the care you receive today. However, talking about your experiences of an event can stir up emotions and touch on sensitive subjects, and if you find it difficult to answer any questions, we will skip them. By participating, you are contributing to increasing knowledge in the field, which can lead to improved healthcare and care in rural areas.

What will happen to my information?

The project will collect and record information about you. In addition to your name and contact details noted during our initial contact with you, the interview will be saved as an audio recording and in text form. The text document will be labelled with a code instead of your name. Only the researcher will be able to link the name to the code during the lifetime of the project. If you would like to see your interview or the results of the entire study, please contact the researcher in charge. It is entirely voluntary whether you choose to see the results of the study or not.

When the project is complete, the recording will be destroyed and no information will be able to be linked to you as a person. The documents will be stored in a locked archive at The Swedish Centre for Rural Health and saved for 10 years in accordance with the rules for storing research data at Swedish universities.

The Swedish Centre for Rural Health, Region Västerbotten, is responsible for your personal data. Under the EU's General Data Protection Regulation, you have the right to access the information about you that is processed in the study free of charge and, if necessary, have any errors corrected. You can also request that information about you be deleted and that the processing of your personal data be restricted. However, the right to erasure and restriction of processing of personal data does not apply when the data is necessary for the research in question. If you wish to access the data, please contact the responsible researcher or data protection officer. Contact details can be found at the bottom of the document. If you are

dissatisfied with how your personal data is processed, you have the right to submit a complaint to the Data Protection Authority.

Who is responsible for the study?

The principal investigator for the study is Region Västerbotten, and the researcher in charge is Anette Edin-Liljegren, research strategist at The Swedish Centre for Rural Health and affiliated with the Department of Epidemiology and Global Health, Umeå University. The study has been approved by the Swedish Ethical Review Authority. If you have any questions, wish to withdraw your consent or wish to access the information collected, please contact the principal investigator or data protection officer below.

Insurance and compensation

The research subjects participating in the study are covered by standard patient injury insurance. No compensation will be paid for participation.

Contact

**Principal investigator:**<br>Anette Edin-Liljegren	**Visiting address:**<br>**Mail:**<br>**Telephone:**
**Data protection officer**<br>Roland Gustavsson	**Visiting address:**<br>**Mail:**<br>**Telephone:**

Appendix 2. Information letter

Consent to participate in study

I have received written information about the study and will have the opportunity to ask questions verbally.

I may keep the written information.

☐ I agree to participate in the study “Different perspectives on digital healthcare appointments – What role does technology play in person-centred care?”

☐ I agree to my data being processed in the manner described in the research participant information.

**Location**	**Signature**
**Date**	**Name**
