# Supplementary material for: Long-Term Care Residents’ Experiences of Telemedicine – Video Consultations With Their Regular Physician in Rural Northern Sweden
Source: J Prim Care Community Health. 2026 Aug 18;17:21501319261477002. doi: 10.1177/21501319261477002 (PMC13487111; doi:10.1177/21501319261477002)
Supplement: Supplementary file 3 [file sj-pdf-3-jpc-10.1177_21501319261477002.pdf]

Appendix 3. Interview guide

Background questions:

How old are you?

What gender do you identify yourself as?

In what country were you born and raised?

Are you familiar with digital solutions (such as social platforms, Skype, Bank-ID, video-calls)

1. What do you think about seeing your physician via video consultation/computer? Can you tell me about the meeting?

Was it easy to get contact with your physician over the screen?

Is it important that the staff know you from before?

Do you think there is anything that works better with this approach than the traditional one?

Do you think there is anything that works less well with this approach than the traditional one?

2. How would you like to be involved in your care?

How would you like to be involved in planning your care?

3. What do you think you can contribute to the care meeting?

How would you like to participate in your care meeting?

What is important for you to contribute to the meeting?

Why?

How do you think this affects the safety and quality of your care?

4. How would you like to receive information about your health and care? Please explain.

How does it work today?

How would you like it to work?

Is there a difference between planned and emergency care appointments?

5. Do you feel safe here in your accommodation? Try to describe.

Appendix 3. Interview guide

Can you tell us about what makes you feel safe or unsafe?

Why do you think that was?

6. What do you consider to be good quality care?

Why is that important?

Interviewer: Summarise the questions. Is there anything else you would like to add? Thank you!

Translated from Swedish with help from DeepL.com (free version)
